# Immunohistochemical expression of ER-α and PR in papillary thyroid carcinoma

**DOI:** 10.3332/ecancer.2017.748

**Published:** 2017-06-13

**Authors:** Marwa Mohammed Serag Eldien, Asmaa Gaber Abdou, Tarek Rageh, Eman Abdelrazek, Enas Elkholy

**Affiliations:** 1 Department of Pathology, Faculty of Medicine, Menoufia University, Gamal Abd-Elnaser street, Shebein Elkom 32511, Egypt; 2 Department of Surgery, Faculty of Medicine, Menoufia University, Gamal Abd-Elnaser street, Shebein Elkom 32511, Egypt; 3 Department of Oncology, Faculty of Medicine, Menoufia University, Gamal Abd-Elnaser street, Shebein Elkom 32511, Egypt

**Keywords:** papillary thyroid carcinoma, ER, PR, immunohistochemistry

## Abstract

Papillary thyroid carcinoma (PTC) is the most common thyroid cancer with multiple risk factors including exposure to ionising radiation. Oestrogens contribute to papillary carcinoma development by promoting cell proliferation and invasion of mutated epithelial follicular cells. The present study aimed to assess ER-α and PR expression in PTC and to correlate their expression with the clinicopathological parameters in this cancer. This study included 62 primary and six metastatic papillary thyroid carcinoma cases. Nineteen and 38.7% of primary PTC cases showed positive nuclear expression for ER and PR, respectively. Metastatic cases showed 66.7% positive ER expression and all were negative for PR. Oestrogen receptor expression showed significant higher positivity in metastatic compared to primary PTC (*p* = 0.02) and it was significantly associated with primary PTC associated with thyroiditis (*p* = .002). Progesterone receptor expression was significantly associated with old age in primary PTC (*p* = .003) and it showed significant coparallel expression with ER (*p* = .000). Oestrogen and progesterone receptors expressed in papillary thyroid carcinoma opening the door for further studies to verify if those patients could benefit from hormonal therapy. Oestrogen receptor seems to have a role in metastatic process of PTC as malignant cells express it in metastatic more than primary site. The presence of lymphocytes in the stroma may promote ER expression in adjacent PTC, necessitating further studies on PTC cases associated with Hashimoto thyroiditis to verify this assumed relationship.

## Introduction

Thyroid cancer is the most common cancer of the endocrine system [1, 2]. It is classified into three main histologic types: differentiated (including papillary, follicular and hurthle), medullary, and anaplastic (aggressive undifferentiated tumour) [3]. Papillary thyroid carcinoma (PTC) represents 80.3% of malignant endocrine tumours and 65% of malignant thyroid tumours [1, 4].

Established risk factors for thyroid cancer are few, including exposure to ionising radiation and some uncommon familial syndromes [5]. Epidemiological data report a strong female predilection for thyroid cancer. The gender ratio of PTC in female and male patients declines from > 5 at 20‑24 years old to 3.4 at 35–44 years old, and reaches almost 1 at > 80 years old, indicating that PTC occurs predominantly during the reproductive years [6]. Female predominance of thyroid cancer in childbearing period suggests that oestrogen and progesterone may play vital roles in the pathogenesis of thyroid neoplasms [7, 8]. Women at menopause were reported to have a reduced thyroid cancer risk [9].

Experimental studies have demonstrated that oestrogens contribute to papillary carcinoma development by promoting cell proliferation and invasion of mutated epithelial follicular cells [8, 10].

Oestrogen and progesterone act through the oestrogen receptor (ER) and progesterone receptor (PR), respectively. Both belongs to the nuclear hormone receptor superfamily and expressed in benign and malignant thyroid tissues. There are two ER isoforms: ER-α, and ERβ. PR has two isoforms, PR‑A and PR‑B [11].

Oestrogen receptor-α and ER-β have opposing effects on cell survival and proliferation. Oestrogen receptor-α has proliferative and antiapoptotic activity, while ER-β shows differentiative and pro-apoptotic effects [12].

Generally, PTC has a favourable long-term prognosis. However, up to 10% of patients with PTC die as a direct result of this carcinoma, and 22–30% experience recurrent disease [13]. It can metastasise to neck lymph nodes in 20–90% of the patients [14]. Distant metastasis is the most common cause of death [15]. Thus, it is important to be able to identify the PTC patients carrying poor prognostic features to select an appropriate treatment and improve the prognosis [16].

The present study aimed to assess ER-α and PR expression in PTC and to correlate their expression with the clinicopathological parameters in this cancer.

## Materials and methods

This study included 68 archival papillary thyroid carcinoma cases (62 primary and 6 cases metastatic to lymph node). The cases were diagnosed in Pathology Department, Faculty of Medicine, Menoufia University in the period between December 2012 and January 2016. Inclusion criteria were the availability of tissue blocks and clinical data. The parameters studied retrieved from pathology records and by the re-evaluation of haematoxylin and eosin-stained slides including patient age, gender, type of operation, tumour size, TNM staging, lymph node involvement, histologic variant of PTC and associated pathology in thyroid tissue away from tumour. The patients were submitted to surgical treatment in the form of total thyroidectomy in 36 cases and hemithroidectomy in 26 cases. Radioactive iodine therapy was further given in Oncology Department, Menoufia University for advanced T3 or T4 tumours, or cancers that have spread to lymph nodes or distant sites. Patients were advised to eat a low-iodine diet for 1–2 weeks before receiving radioactive iodine therapy (131 I). Patients who finished radioactive iodine treatment and those who were treated by total thyroidectomy received daily thyroid hormone (levothyroxine) and followed every 6 months by neck ultrasound.

### Immunohistochemistry

From each case, multiple 4-µm-thickness sections were cut (1 for each primary antibody). The slides were subjected to subsequent steps of deparaffinisation and rehydration. Antigen retrieval was performed by boiling in citrate buffer saline (pH 6) for 20 min, followed by cooling at room temperature. Endogenous peroxidase activity was blocked by incubation with 6% H_2_O_2_ in methanol. The primary antibodies used were mouse monoclonal anti-human oestrogen receptor alpha (ER), clone 1D5 (ready to use) (Dako, Copenhagen, Denmark) and mouse monoclonal anti-human progesterone receptor (PR), clone PgR 636 (ready to use) (Dako, Copenhagen, Denmark). The primary antibodies were incubated overnight at room temperature. Immunoreactivity for ER and PR was visualised using Envision system (Dako, Copenhagen, Denmark) with DAB chromogen as substrate and Mayer’s haematoxylin as counterstain. ER-positive breast carcinoma and PR-positive breast carcinoma were used as positive control for ER-alpha and PR, respectively. For all reagents, negative controls were prepared by substituting the primary antibodies with cross-matched isotopes.

### Interpretation of immunostaining

Nuclear staining in any number of malignant cells is required to assign ER and PR positivity.

### Statistical analysis

Data were collected, tabulated, and statistically analysed using a personal computer with the ‘statistical package for the social sciences’ (SPSS) version 23. The *χ*^2^ and the Fisher exact tests used for comparison between qualitative variables. The Mann–Whitney U-test was used for comparison between qualitative and quantitative variables.

*p* ≤ 0.05 considered significant.

## Results

### Clinicopathological Data

The clinical and pathological data are shown in Table 1.

### Oestrogen and progesterone receptor expression in primary and metastatic cases (Table 2)

**Figure 1.**
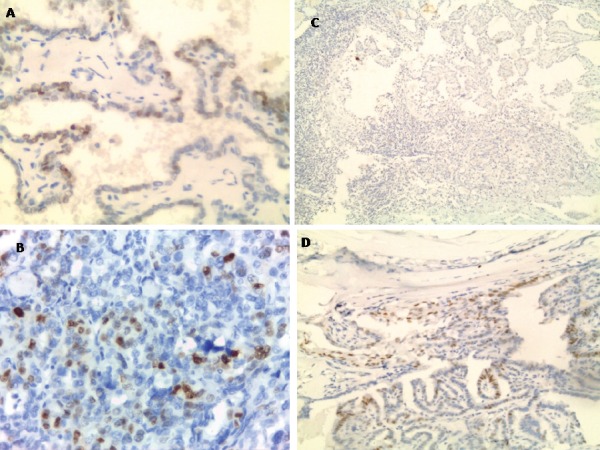
Nuclear ER expression in primary classic papillary thyroid carcinoma (A) and follicular variant papillary thyroid carcinoma (B) (IHC x400). Negative PR expression in metastatic papillary thyroid carcinoma to lymph node (C) compared to positive expression in primary papillary thyroid carcinoma (D) (IHC x100)

Twelve out of 62 (19.3%) primary PTC cases were positive for ER (Figure 1A and B) in comparison to four out of six (66.7%) metastatic cases with a statistically significant difference (*p* = 0.02). Regarding PR, all metastatic and 61.3% (38/62) of primary cases were negative for PR (Figure 1C). The difference was not significant (*p* = 0.08). PR showed nuclear expression in 38.7% of primary PTC (24/62) (Figure 1D).

### Association between ER expression and clinicopathological data of studied cases (Table 3)

Positive ER expression showed a highly significant association with the presence of thyroiditis in thyroid tissue away from tumour (*p* = 0.002).

No significant association was noticed between ER expression and other clinicopathological data including age, sex, tumour size, histological subtype, stage, nodal status, and focality.

### Association between PR expression and clinicopathological data of studied cases (Table 4)

PR expression was significantly associated with age of PTC patients (*p* = 0.003), since median age in cases negative for PR was 37 years, compared to 55 years in cases positive for PR.

On the other hand, no significant association was noticed between PR expression and other clinicopathological data.

### Relationship between ER and PR expression

**Figure 2.**
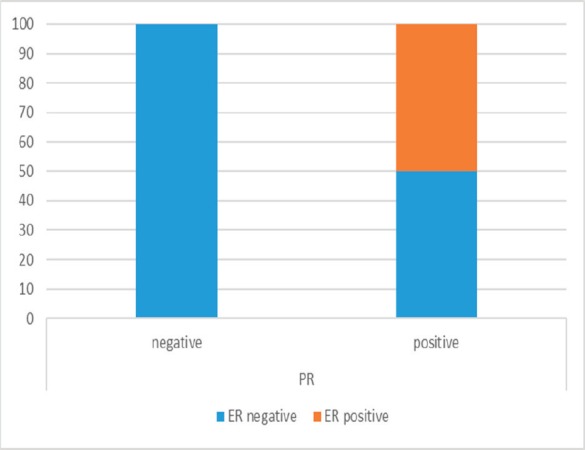
All cases negative for PR were simultaneously negative for ER.

There was a significant coparallel expression of ER and PR in malignant cells (*p* = 0.000), that is, all cases negative for PR were also negative for ER (Figure 2).

### Follow-up data

Follow-up data were available for only five patients, three of them were stage T1 and all were negative for ER with only one case was positive for PR and they were free of the disease in the last visit (December, 2016). Those patients were treated only by surgical management (total thyroidectomy). The fourth patient was T1 stage who underwent total thyroidectomy but follow-up detected lymph node metastasis that necessitated radioactive iodine therapy. The fifth patient was T2 stage who underwent left hemithyroidectomy and follow-up detected recurrence in right side, which required completion of thyroidectomy and receiving radioactive iodine therapy. The carcinomas of the fourth and fifth cases were positive for both ER and PR.

## Discussion

The expression pattern of ER isoforms has been demonstrated in neoplastic and non-cancerous human thyroid tissues; however, the results are not consistent [17, 18].

In the current study, we confirmed that both ER-α and PR were expressed in PTC cells as observed in many studies [19–21]. In addition, several different thyroid cancer cell lines have been shown to express ER and PR [10, 16, 22, 23].

In the present study, the percentage of cases positive for PR (38.7%) were more than the percentage of cases positive for ER-α (19.3%). Some studies found the same results [20, 21, 24].

There is an increasing number of studies indicating that oestrogen may exert a direct effect on tumorigenesis in human thyroid cells by ER-dependent or ER-independent mechanisms through modulating cell proliferation, modulation of sodium‑iodide symporter and thyroglobulin gene expression [16, 17, 18, 25]. The proliferative effects of 17β-oestradiol (E2) in thyroid cancer were found to be mediated through the regulation of genes involved in growth control, such as bcl-2, Bax, and c-fos [10, 26]. The proliferation of these cells was stimulated by ER-α agonists, and downregulated by ER-β agonists [27].

In the present study, metastatic PTC cases showed significant higher ER expression than primary cases. Vannucchi *et al*., (2015) [21] observed that there is a tendency to higher incidence of local metastasis in ER- and PR-expressing tumours. Many experimental studies have found that E2 could induce metastatic potential of several PTC cell lines by enhancing adhesion, migration, and invasion of cells [8, 16, 28].

The metastatic process requires cancer cells to leave the primary tumour and to acquire migratory and invasive capabilities. Many different processes are involved such as epithelial to mesenchymal transition (EMT), adhesion molecules downregulation, and matrix metalloproteinases (MMPs) upregulation in cancer cells. Loss of epithelial protein marker E-cadherin, the concurrent upregulation of mesenchymal protein markers vimentin, and the upregulation of MMP-9 play a dominant role in the metastatic process and could be regulated by E2 in various cancers, including breast, ovarian, colon, and lung cancer [29–31].

Oestradiol has been found to increase the metastatic potential of human epithelial ovarian cancer cell through the upregulation of MMP-2 and downregulation of E-cadherin [29, 32]. Oestradiol enhanced breast cancer cell motility and invasion via extranuclear activation of actin-binding protein ezrin [30]. Oestradiol promoted lung cancer cell migration through downregulation of E-cadherin and *β*-catenin and upregulation of fibronectin and vimentin [33].

Oestradiol induces endothelial proliferation and migration mediated by the classic oestrogen receptor, which is expressed by endothelial cells. This effect provides a potential mechanism contributing to the angiogenic effect of oestradiol [34–36]. This may be a possible explanation for the higher expression of ER-α in metastatic cases.

Park *et al*., (2008) [29] found that E2 triggered the metastatic behaviours exclusively through an ER-α-dependent pathway, but ER-β had an opposing action on ER-α in ovarian cancer. Many studies have concluded that the expression ratio between ER-α and ER-β determines the direction of response to oestrogens [27, 37, 38]. Thus, ER-α- and ER-β-selective agonists are promising new approach for treating specific conditions associated with endocrine-related disease [25].

Immunohistochemical evaluation of ER-α expression in patients with PTC may present a potential prognostic marker [19] and possibly indicates a more aggressive behaviour agreeing with our limited follow-up where the cases that showed recurrence and metastasis to lymph node were originally positive for ER. Moreover, it will have profound biological and therapeutic implications and could modify the follow-up, particularly in fertile women affected with persistent disease [16, 21].

In this study, the presence of inflammatory cells in thyroid tissue away from tumour was significantly associated with ER-α expression. The assumed relation between E2 and inflammatory response may explain this observation. Oestradiol has a complex immunomodulatory effect upon inflammation. Oestradiol suppresses acute lung inflammatory responses of mice through an effect on vascular cell adhesion molecules and proinflammatory mediators [39]. However, E2 can also have a pro-inflammatory role [40]. Oestradiol can stimulate T-cell-dependent immune responses [41].

There was no significant association between ER-α expression and other studied clinico-pathological parameters including age, sex, histological subtype, stage, nodal status, and focality agreeing with others [19, 21]. On the other hand, significantly elevated expression of ER-α was identified in PTC patients with a large tumour size [19, 21, 24].

Regarding PR, PTC-positive cases showed only significant association with older age at presentation and showed significant coparallel expression with ER-α agreeing with Struniolo *et al*. [24].

## Conclusions

The results of the present study indicate that malignant cells in PTC express both ER and PR, opening the door for further studies to verify whether those patients could benefit from hormonal therapy. Oestrogen receptor seems to have a role in the metastatic process of PTC, as it is expressed in the metastatic more than the primary site. The presence of lymphocytes in the stroma may promote ER expression in adjacent PTC, necessitating further studies on PTC cases associated with Hashimoto thyroiditis to verify this assumed relationship.

## Figures and Tables

**Table 1.** Clinicopathological data of studied papillary thyroid carcinoma cases.

Primary cases (62)				Metastatic cases (6)	
		No.	%	No.	%
***Age***	*mean* ± *SD*	45.5 ± 15.7		31.3 ± 20.2	
	*Median*	47		29	
	*Range*	18–70		10–55	
***Sex***	*Male*	20	32.3	6	100
	*Female*	42	67.7	0	0
	*M:F ratio*	1:2.1			
***Specimen***	*Right hemithyroidectomy*	22	35.5		
	*Left hemithyroidectomy*	4	6.5		
	*Total hemithyroidectomy*	36	58.1		
***Size***	*Mean* ± *SD*	2.5 ± 1.9			
	*Median*	1.8			
	*Range*	0.2–7			
***Histological subtype***	*Classic*	32	51.6	6	100
	*Follicular variant*	12	19.4	0	0
	*Microcarcinoma*	18	29	0	0
***Stage***	*T1*	34	54.8		
	*T2*	16	25.8		
	*T3*	12	19.4		
***Lymph node***	*negative/not assessed*	46	74.2		
	*Positive*	16	25.8		
***Focality***	*Single*	50	80.6		
	*Multifocal*	12	19.4		
***Background***	*Multinodular*	52	83.9		
	*Thyroiditis*	10	16.1		

**Table 2.** Expression of ER and PR in primary and metastatic PTC cases.

		Primary	Metastatic	Test	p value
***ER***	*Negative*	50 (80.7%)	2 (33.3%)	FE = 6.8	***0.02 S***
	*Positive*	12(19.3%)	4(66.7%)		
***PR***	*Negative*	38(61.3%)	6(100%)	FE = 3.6	0.08
	*Positive*	24(38.7%)	0(0%)		

**Table 3.** Association between ER and studied clinicopathological data.

Primary cases		ER		Test	p value
		negative (50)	positive (12)		
***Age***	*Mean* ± *SD*	44.7 ± 16	48.8 ± 14.7	U = 254	0.4
	*Median*	45	55		
	*Range*	18–70	29–68		
***Sex***	*Male*	18 (90)	2 (10)	FE = 1.7	0.3
	*Female*	32 (76.2)	10 (23.8)		
***Specimen***	*Hemithyroidectomy*	22 (84.6)	4 (15.4)	FE = 0.5	0.5
	*Total hemithyroidectomy*	28 (77.8)	8 (22.2)		
***Size***	*mean* ± *SD*	2.4 ± 1.8	2.9 ± 2.5	U = 262	0.5
	*Median*	1.8	2.2		
	*Range*	0.2–6	0.5–7		
***Type***	*Classic*	26 (81.2)	6 (18.8)	X^2^ = 0.2	0.9
	*Follicular variant*	10 (83.3)	2 (16.7)		
	*Microcarcinoma*	14 (77.8)	4 (22.2)		
***Stage***	*T1*	28 (82.4)	6 (17.6)	X^2^ = 2	0.4
	*T2*	14 (87.5)	2 (12.5)		
	*T3*	8 (66.7)	4 (33.3)		
***Lymph node***	*Negative/not assessed*	36 (78.3)	10 (21.7)	FE = 0.6	0.7
	*Positive*	14 (87.5)	2 (12.5)		
***Focality***	*Single*	42 (84)	8 (16)	FE = 1.9	0.2
	*Multifocal*	8 (66.7)	4 (33.3)		
***Background***	*Multinodular*	46 (88.5)	6 (11.5)	FE = 12.6	***0.002HS***
	*Thyroiditis*	4 (40)	6 (60)		

**Table 4.** Association between PR and studied clinicopathological data.

Primary cases		PR		Test	p.value
		negative (38)	positive (24)		
***Age***	*Mean* ± *SD*	40.7 ± 15.7	53.1–12.6	U = 254	***0.003HS***
	*Median*	37	55		
	*Range*	18–70	29–68		
***Sex***	*Male*	14 (70)	6 (30)	FE = 0.9	0.3
	*Female*	24 (57.1)	18 (42.9)		
***Specimen***	*Hemithyroidectomy*	18 (69.2)	8 (30.8)	FE = 1.2	0.3
	*Total hemithyroidectomy*	20 (55.6)	16 (44.4)		
***Size***	*Mean* ± *SD*	2.4 ± 1.8	2.8 ± 2.1	U = 400	0.4
	*Median*	1.8	2.3		
	*Range*	0.2–6	0.2–7		
***Histological subtype***	*Classic*	16 (50)	16 (50)	X^2^ = 4.4	0.1
	*Follicular variant*	10 (83.3)	2 (16.7)		
	*Microcarcinoma*	12 (66.7)	6 (33.3)		
***Stage***	*T1*	22 (64.7)	12 (35.5)	X^2^ = 0.8	0.7
	*T2*	10 (62.5)	6 (37.5)		
	*T3*	6 (50)	6 (50)		
***Lymph node***	*Negative/not assessed*	28 (60.9)	18 (39.1)	FE = 0.01	0.9
	*Positive*	10 (62.5)	6 (37.5)		
***Focality***	*Single*	30 (60)	20 (40)	FE = 0.2	0.8
	*Multifocal*	8 (66.7)	4 (33.3)		
***Background***	*Multinodular*	34 (65.4)	18 (34.6)	FE = 2.3	0.2
	*Thyroiditis*	4 (40)	6 (60)		
